# Turning a Targeting β-Catenin/Bcl9 Peptide Inhibitor into a GdOF@Au Core/Shell Nanoflower for Enhancing Immune Response to Cancer Therapy in Combination with Immune Checkpoint Inhibitors

**DOI:** 10.3390/pharmaceutics14061306

**Published:** 2022-06-20

**Authors:** Weiming You, Fang Ma, Zhang Zhang, Jin Yan

**Affiliations:** 1 National & Local Joint Engineering Research Center of Biodiagnosis and Biotherapy, The Second Affiliated Hospital of Xi’an Jiaotong University, Xi’an 710004, China; youweiming1014@xjtu.edu.cn (W.Y.); mf0826@mail.xjtu.edu.cn (F.M.); 2 Department of Tumor and Immunology in Precision Medical Institute, Western China Science and Technology Innovation Port, Xi’an 710004, China; 3 General Surgery Department, Tang Du Hospital, Fourth Military Medical University, Xi’an 710032, China

**Keywords:** immunotherapy, drug delivery, β-catenin, Bcl9 peptide, nanotechnology

## Abstract

Combination administration is becoming a popular strategy in current cancer immunotherapy to enhance tumor response to ICIs. Recently, a peptide drug, a protein–protein interaction inhibitor (PPI), that disrupts the β-catenin/Bcl9 interaction in the tumoral Wnt/β-catenin pathway has become a promising candidate drug for immune enhancement and tumor growth inhibition. However, the peptide usually suffers from poor cell membrane permeability and proteolytic degradation, limiting its adequate accumulation in tumors and ultimately leading to side effects. Herein, a gadolinium–gold-based core/shell nanostructure drug delivery system was established, where Bcl9 was incorporated into a gadolinium–gold core–shell nanostructure and formed GdOFBAu via mercaptogenic self-assembly. After construction, GdOFBAu, when combined with anti-PD1 antibodies, could effectively inhibit tumor growth and enhance the response to immune therapy in MC38 tumor-bearing mice; it not only induced the apoptosis of cancer cells, but also promoted the tumor infiltration of Teff cells (CD8^+^) and decreased Treg cells (CD25^+^). More importantly, GdOFBAu maintained good biosafety and biocompatibility during treatment. Taken together, this study may offer a promising opportunity for sensitizing cancer immunotherapy via metal–peptide self-assembling nanostructured material with high effectiveness and safety.

## 1. Introduction

The therapeutic outcomes of immune checkpoint inhibitors (ICIs), such as anti-PD1 and anti-CTLA4, have garnered great attention [1,2,3]. However, with the wide-ranging clinical application of ICIs, the immune response to drugs in cancer patients is becoming a predominant hindrance to immunotherapy, as only a minority of patients can benefit from immune checkpoint blockade (ICB) therapy [4,5]. Mechanistic studies indicated that this hypersensitivity is closely related to the immunosuppressed tumor microenvironment, which attenuates the infiltrating capacity and activity of Teff and the recruitment of DCs, resulting in immune evasion [6,7]. Thus, there is, indeed, an urgent medical need to develop therapeutic agents that boost cancer sensitivity to ICs in immunotherapy.

Numerous published studies have demonstrated that the activation of the Wnt/β-catenin pathway positively correlates with the immune evasion emerging in cancer [8,9,10,11]. For example, in the immunohistochemical analysis of CRC, the overexpression of β-catenin in cancer significantly reduced the tumor infiltration of CD8^+^ T cells, considered key effectors of immune cells in anti-tumor immunity. Moreover, TCGA analysis showed that it was enriched in non-T-cell-inflamed tumors [12]. In the metastatic melanoma model, the tumor-intrinsic hyperactive β-catenin could elicit T cell exclusion and ICI therapy resistance [13]. These findings provide a strong rationale for the development of bioactive inhibitors that target this pathway with immune checkpoint blockades in order to overcome resistance and enhance immunotherapy [14,15,16,17].

β-catenin is usually considered an “undruggable” target as it lacks a deep hydrophobic pocket, making it hard for the inhibitors to directly bind β-catenin for therapeutic cancer interventions [18]. Additionally, the effective inhibition of relevant targets of the upstream pathway can simultaneously impair the development of normal cells and elicit substantial side effects [19,20]. Thus, targeting the β-catenin-associated downstream pathway partners is a potential alternative approach. Bcl9 (B-cell/Lymphoma-9), a coactivator of nucleus β-catenin in the downstream pathway, is reported to convey β-catenin to TCF and promote the expression of Wnt-responsive transcriptional genes (e.g., c-Myc, cyclinD1), many of which are closely related to tumorigenesis and malignant progression [21,22]. Additionally, Bcl9 is overexpressed in carcinoma, and its deletion can safely suppress tumorigenesis and invasion in β-catenin-dependent cancer models [23,24,25,26]. More importantly, unlike most β-catenin partners (TCF, E-cadherin) that occupy the same binding site and cause off-target and severe toxicity in clinical trials, Bcl9 only adopts α-helix to bind the first arm repeat of β-catenin and barely affects the interaction of other partners with β-catenin [27]. Given these factors, many efforts have been made for the development of bioactive peptides that block the β-catenin/Bcl9 interaction in cancer therapy, for instance, hsBCL9CT-24 [28], sulfono-γ-AA [29], and ECRV [30]. Nevertheless, no β-catenin/Bcl9 inhibitors have been approved for clinical use. Reasons for failure are as follows: poor cell membrane permeability and susceptibility to proteolytic degradation [31,32]. Hence, developing a safe and effective platform that can deliver peptides to the intracellular target is necessary. Nanomedicine has arisen as a powerful tool to address these obstacles [17,33,34]. Despite some success in some nanoformulations [35,36], such as a Gd–Au-based nanohybrid, there is still an enormous challenge in clinical translation due to the complicated synthetic process, diverse building blocks, and high cost. Thus, the selection of nanoformulations that are simple, easily scalable, and compatible with medicine is necessary. 

**Figure 1:**
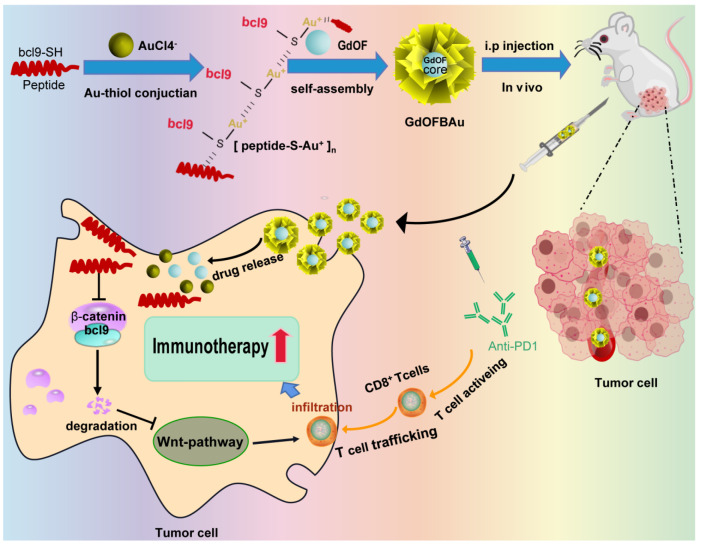
Schematic diagram of the preparation of GdOFBAu and its action.

Herein, we developed a mild and straightforward process to prepare a gadolinium–gold-based core–shell nanohybrid via a one-pot, two-step method. Lanthanide-doped nanocrystals (LDN) have higher coordination numbers than conventional inorganic nanoparticles, such as gold, silver, and platinum nanoparticles [37,38]. Gd can conjunct with Au multiple times. Moreover, the Au–Gd bond is much stronger compared to an Au–Au bond [39,40]. Thus, GdOF was used as core material in this paper to optimize loading capacity. To overcome the pharmaceutical obstacle of peptides in sensitizing cancer immunotherapy, Bcl9 peptides bridged Au^1+^ ions via Au–s bonds in a reducing environment to form a peptide Au precursor polymer as a shell which then self-assembled with GdOF to produce GdOFBAu via metal–organic coordination (Figure 1). In this way, GdOFBAu could successfully deliver Bcl9 peptides to MC38 tumor-bearing mice to inhibit tumor growth and increase the response of mice against anti-PD1 while exhibiting favorable biosafety and biocompatibility at the same time. To sum up, the therapeutic regimen adopted in this study may provide a new referential experience for combination administration in immunotherapy.

## 2. Experimental Materials and Methods

### 2.1. Reagents Information 

All L-amino acid raw materials used for synthesis in the experiment were purchased from C S Bio (Shanghai, China) Ltd. Hexahydro pyridine (Piperidine, AR) was purchased from Sinopharm, and Rink-amide MBHA resin (loading: 0.46 mmol/g) was ordered from Tianjin Nankai Hecheng (Tianjin, China). Other reagents were purchased from Sigma (St. Louis, MO, USA) unless otherwise specified.

### 2.2. Solid-Phase Chemical Synthesis of Anti-Tumor Peptide Bcl9

The entire synthetic reaction process for Bcl9 peptide was performed in a reaction vessel by Cs136XT Peptide Synthesize, where it was incorporated into swelled resin from C-terminal to N-terminal, and the detailed reaction program was set up as previously reported [41]. Subsequently, the crude peptide was isolated from Rink-amide MBHA resin in a corrosion-resistant glass containing the TFA cleavage cocktail (88% TFA, 5% phenol, 5% deionized water, 2% Tips) at the end of synthesis. After stirring for 2 to 3 h, it was precipitated three times with ice-cold ether and dried and purified via semi-preparative reversed-phase C18 column (10 μm, 120 A, 250 nm × 50 nm) using a gradient elution of water/acetonitrile. Lastly, the identification of the molecular weight of the desired product was characterized by Waters 2695 ESI-MS (*m*/*z*:400~1600) under the positive charge model.

### 2.3. Preparation of GdOFBAu

The synthesis of GdOFBAu proceeded mildly with a one-pot, two-step method for assembling the GdOF nanocore with polypeptide–gold polymer nanoshell. GdOF was produced as previously reported [42]. Specifically, 2 mg Bcl9 peptide was added into a 50 mL clean beaker containing 2.25 mL NH2-PEG-SH (2 mg, MW:2K) ethanol–aqueous solution. After complete dissolution of the peptide, 2.25 mL 50 mM PH 7.0 aqueous HEPES (4-(2-hydroxyerhyl) piperazine-1-erhanesulfonic acid) solution plus 500 μL 10 mM HAuCl_4_ aqueous solution were added to form a polypeptide–gold polymer nanoshell (5 min, 500 rpm, 50 °C). Meanwhile, in another 50 mL clean beaker, 2.25 mL 50 mM PH 7.0 aqueous HEPES was mixed with 2.25 mL GdOF solution (0.45 mg dispersed in 20% acetonitrile and 80% standard PBS) and 500 μL 10 mM HAuCl_4_ aqueous solution. After stirring for 5 min, it was mixed with polypeptide–gold polymer nanoshell at 50 °C, 300 rpm, for 10 min and then the GdOFBAu was prepared and dried for further use.

### 2.4. Characterization of Physicochemical Properties of GdOFBAu

#### 2.4.1. HRETEM of GdOFBAu

Copper-grid-coated carbon film (300 mesh, Beijing, Electron Microscopy China) was picked up via tweezers and placed face up in a Petri dish lined with filter paper. Then, 10 μL GdOFBAu solution was dropped onto copper grids. After drying at room temperature, the copper grid was put into the HRTEM holder, and the morphology of GdOFBAu was assessed using a Lorenz Transmission Electron Microscope (Thermo Fisher, Waltham, MA, USA, Talos F200X) at 200 kV.

#### 2.4.2. FESTEM of GdOFBAu

Doubled-sided conductive tape was stuck to the SEM table, then a little GdOFBAu powder was attached to conductive tape. After the loose powder was gently blown off by a suction ear bulb, the SEM table was put into FESEM microscope (FEI, Quanta 250FEG, Hillsboro, OR, USA), and the morphology of GdOFBAu was observed at 20 kV.

#### 2.4.3. FT–IR of GdOFBAu

A 1 mg amount of dried GdOFBAu powder was mixed with 120 mg dried KBr powder, and the mixture was pressed into a thin disc with the tableting machine for Brooke VERTEX 70 microscopic infrared spectrometer analysis (wavelength range: 400–4000 cm^−1^, scanning speed: 1 s, number of scans: 32).

#### 2.4.4. UV–Vis of GdOFBAu

A 3 mL quantity of the liquid sample was loaded into a clean quartz cuvette (capacity: 3.5 mL, optical path: 10 mm) and placed into the sample room at room temperature. The UV spectrum was scanned at core facilities sharing platform by a Lambda 950 UV–Vis–NIR spectrophotometer (wavelength range: 200–900 nm, scanning step: 0.5 nm).

#### 2.4.5. Granularity and Zeta Potential of GdOFBAu

A 1 mL quantity of the liquid sample was transferred into a clean quartz cuvette (optical path: 10 mm) for granularity (range: 10–500 nm, scanning step: 5.3 nm) or universal dip cell for zeta potential (range: −150 mV–150 mV, scanning step: 3 mV). The granularity and zeta potential were recorded by Zetasizer Nano ZS.

#### 2.4.6. XPS of GdOFBAu

Doubled-sided conductive tape was stuck to the sample table, then 2 mg of sample powder was attached to the conductive tape. The sample table was put into the XPS spectrometer (Thermo Fisher, Massachusetts, USA, ESCALAB Xi^+^). The test conditions of high-resolution element narrow spectrum: X-ray: 500 μm, pass energy: 30 eV, step size: 0.1 eV.

#### 2.4.7. XRD of GdOFBAu

A 10 mg amount of sample powder was spread on a glass slide. After the glass slide was placed into the sample holder, the XRD was obtained by using Bruker D8 Advance X-ray diffractometer (voltage: 40 kV, current: 40 mA, step size: 0.02, time per step: 1 s, 2θ range: 20°–90°).

#### 2.4.8. Raman of GdOFBAu

A 2 mg amount of sample powder was spread on a glass slide. After the glass slide was placed into the microscope stage, the Raman was conducted on a Renishaw in Via Qontor Laser Raman spectrometer by 785 nm line laser (wavelength range: 95–3200 cm^−1^, exposure: 400 ms, objective: 10×, laser power: 250 mw).

### 2.5. Cell Culture, Apoptosis, Cell Cycle, and Western Blot Analysis

#### 2.5.1. Cell Culture

The MC38 cell line was obtained from the Chinese Academy of Sciences Cell Bank and grown at 37 °C in a humidified incubator with 5% CO_2_. For 48 h, cells were plated on the sterile 96-well plates (400 cells/well) with the indicated treatment. The vitro cytotoxicity of medication was evaluated using an automatic microplate reader at 450 nm after adding CCK8 reagent to microtiter wells for 4 h.

#### 2.5.2. Cell Apoptosis

For cell apoptosis analysis, fresh cells were plated in the sterile 6-well Petri dishes containing DMEM medium (500,000 cells/well) for 48 h and then treated with control (PBS), GdOFAu, and GdOFBAu, respectively. After 48 h of incubation, the cells were harvested in the EP tube and washed twice with the cold PBS; collected cells were resuspended in 1x staining buffer at a concentration of 1 × 10^6^ cells/mL. After the addition of Annexin V-FITC and PI, the cells were gently resuspended with a pipette and incubated on ice for 10 min without light.

#### 2.5.3. Cell Cycle

In the cell cycle, cells were plated in sterile 6-well Petri dishes containing DMEM medium (500,000 cells/well) for 48 h and then treated with the control (PBS), GdOFAu, and GdOFBAu, respectively. After 48 h of incubation, the cells were harvested in the EP tube and resuspended in 70% ethanol. After being fixed at −20 °C for 4 h, the cells were washed twice using cold PBS. PI, RNase A, and Triton-X 100 (0.2%) were sequentially added, and, after being gently resuspended with the pipette, the cells were incubated on ice for 10 min without light.

#### 2.5.4. Western Blot

The cells incubated with the indicated drugs for the specified times were centrifuged, and the supernatant was discarded with the pipette. The precipitation of cells was lysed by RIPA buffer, followed by BSA quantification, and then the expression of protein was measured by Western blot assay, as previously reported [43,44]. 

### 2.6. Subcutaneous Xenograft Experiment in C57 Mice

A total of 20 female C57 mice, raised in the school SPF animal house, were obtained from Charles River, Beijing, and the experiment was approved by Xi’an Jiaotong University Ethics Committee. A 100 μL amount of fresh MC38 cells (5 × 10^5^ per mice) was subcutaneously inoculated into dorsal flanks of (4~5 weeks) C57 mice. Mice bearing MC38 tumor (Volume = 50 mm^3^) were randomly divided into 4 groups (n = 4): control (PBS), PD1, GdOFBAu, GdOFBAu + PD1. Drug treatment was administered 5 times intraperitoneally every 2 days (PD1 5 mg/kg per mice, GdOFBAu 2 mg/kg per mice). Tumor volume and the weight of mice were documented in the notebook, measured daily by vernier caliper and scales, respectively. Histology and immunostaining were measured as per our previous lab report [45].

### 2.7. The Detection of Biochemical Indicators of Tumor-Bearing Mice Blood

At the end of the experiment, the blood of the tumor-bearing mice was collected into EDTA anticoagulation tubes and sent to the medical laboratory for the detection of biochemical indexes, such as WBC and RBC.

### 2.8. Statistical Analysis 

The mean of the standard deviation (SD) of three independent tests was recorded in this study, and mean standard deviation differences between groups were examined for statistical significance using GraphPad Prism, version 9.1. Other characterization data were analyzed by Origin 2019. 

## 3. Result

### 3.1. Construction and Characterization of GdOFBAu

**Figure 2:**
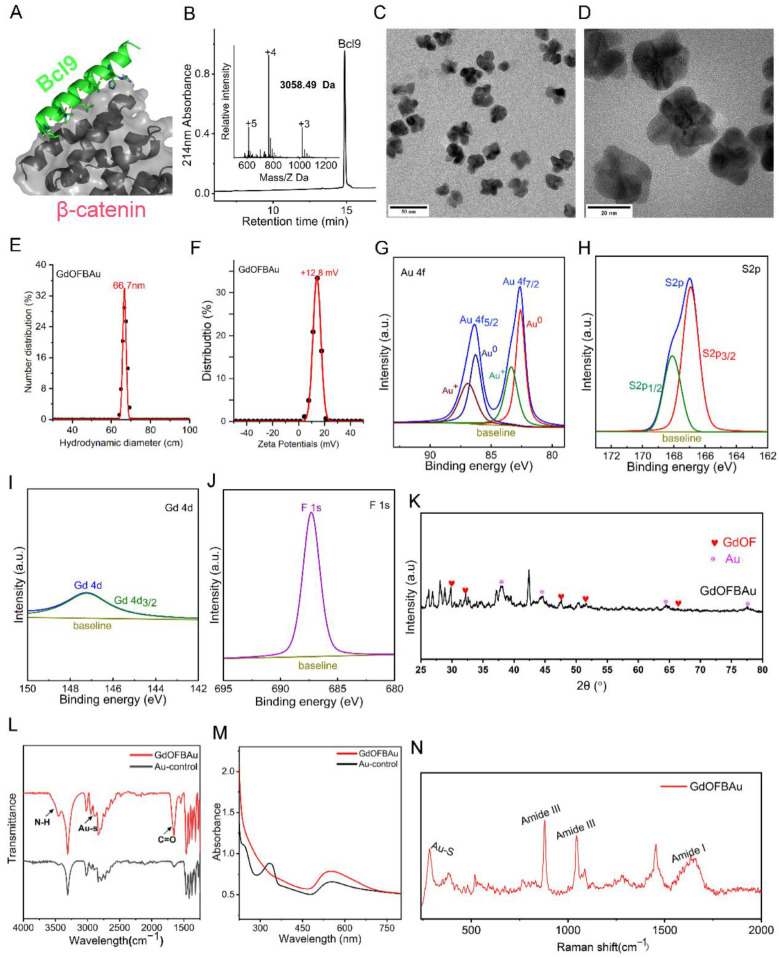
Characterization of GdOFBAu. (**A**) Molecular docking simulated structures of Bcl9 peptide aligned with β-catenin. (**B**) Characterization of Bcl9 by HPLC and ESI-MASS. (**C**,**D**) HRTEM images of GdOFBAu. (**E**) Hydrated particle size distribution of GdOFBAu determined by DLS. (**F**) Zeta potential of GdOFBAu. (**G**–**J**) XPS spectrum of Au (**G**), S (**H**), Gd (**I**), and F (**J**) elements in GdOFBAu. (**K**) XRD of GdOFBAu. (**L**) UV–VIS absorption spectra of GdOFBAu, scan range: 200–900 nm, solvent: deionized water. (**M**) FT–IR spectra of GdOFBAu powder measured by potassium bromide compression. (**N**) Raman spectra of GdOFBAu powder under excitation at 785 nm.

The synthesis of GdOF was produced through the hydrothermal method as previously reported [42]. In our design, an additional mercapto-containing Cys residue was employed at the C-terminal of the peptide sequence for conjugating Bcl9 (a targeting β-catenin peptide, sequence: Ac-SQEQLEHRERSLQTLRDLQRMLFC-NH_2_) to the surface of the monovalent gold ion (Au^1+^), and the peptide was synthesized by standard SPSS using the Fmoc (N-(9-fluorenyl) methoxycarbonyl) chemistry method and characterized by ESI-MS after purification (Figure 2A,B). In the presence of the reducing agent HEPES, Au^3+^, in an aqueous chloroauric acid solution, was reduced, where Au^1+^ ions could conjugate with Bcl9 through an Au–S bond to form a transparent, polymeric Bcl9–Au(I) solution, termed [Bcl9-S-Au^+^]_n._ Subsequently, [Bcl9-S-Au^+^]_n_ self-assembled with GdOF to form the core–shell structured nanohybrid GdOFBAu. After the formulation of GdOFBAu, the transmission electron microscope revealed that it predominantly formed a monodispersed nano-star-like morphology with an average diameter of ~30 nm, a suitable size for tumor accumulation via the EPR effect [46] (Figure 2C,D). The morphology of GdOFBAu according to FESEM image, consisted of a number of small, dispersed particles, consistent with the TEM results (Figure S1). The hydrodynamic diameter of GdOFBAu was observed to be around ~66.7 nm via dynamic light scattering experiments (Figure 2H). This phenomenon may be attributed to the hydrated layer on the surface of GdOFBAu. The zeta potential of GdOFBAu was 12.8 mV, which was more easily taken up by cells (Figure 2F). Moreover, X-ray photoelectron spectroscopy exhibited the composition of Au, Gd, F, and S elements, confirmed by the characteristic electronic energy signals of Au, Gd, F, and S (Figure 2G–J). The presence of Au and GdOF was further proved by XRD data, as previously reported (Figure 2K) [43,47,48]. To further confirm the successful self-assembly of GdOFBAu, FT–IR, UV–vis, and Raman experiments were performed. As shown in Figure 2L, three characteristic absorption peaks of amide bonds (1650 cm^−1^, 3450 cm^−1^) and Au–S (2950 cm^−1^) were observed in GdOFBAu compared to in the Au core, suggesting the successful self-assembly of GdOFBAu. In agreement with this conclusion, a characteristic absorption peak of Au at 550 nm in the UV–Vis spectrum (Figure 2M) and the characteristic peaks of the Au–S and amide bond appeared in the Raman spectra (Figure 2N). 

### 3.2. GdOFBAu Inhibited Tumor Proliferation In Vitro by Suppressing the Wnt/Beta-Catenin Pathway

**Figure 3:**
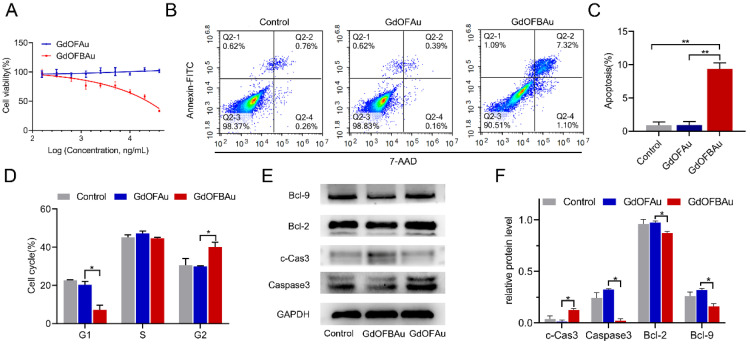
In vitro anticancer efficacy and mechanism of GdOFBAu on MC38 cells. (**A**) MC38 cells were treated with increasing concentrations of drugs, as determined by the MTT assay, after 48 h incubation. (**B**,**C**) Quantitative analysis of the apoptosis of MC38 cells induced by the indicated drugs, as assessed by FACS analysis. (**D**) Cell cycle profiles of MC38 cells by FACS analysis. (**E**,**F**) The protein expression of c-Cas3 (cleaved-Caspase3), Caspase3, Bcl2 and Bcl9 in MC38 cells after drug treatment was measured by Western blot assay. *p*-values were determined by student’s *t*-test: **, *p* ≤ 0.1; *, *p* ≤ 0.5.

In addition to immune evasion, aberrant activation of β-catenin is also crucial for tumorigenesis and malignant progression, which emerges in approximately 80% of colon cancer cases [49]. As per our design, GdOFBAu conferred the ability to target tumors with the peptide Bcl9 and then selectively inhibited tumor cell proliferation and apoptosis by blocking the β-catenin/Bcl9 interaction. To confirm this, we performed an MTT assay on MC38 mouse colon cancer cells. As shown in Figure 3A, we treated the MC38 cells with different doses of drugs, as indicated, for 48 h. The data results showed that GdOFBAu inhibited the cell viability in a dose-dependent, increasing manner, whereas GdOFAu did not affect the cell proliferation. Compared to GdOFAu treatment, GdOFBAu treatment resulted in a statistically significant increase in the apoptosis ratio of MC38 cells in the Annexin V/PI (Figure 3B,C). Consistent with this finding, GdOFBAu resulted in cycle arrest at the G2 phase compared with other groups (Figure 3D). To further investigate the intrinsic mechanism underlying the inhibition of MC38 cells, Western blot was performed. As expected, the level of pro-apoptotic factor c-Cas3 (cleaved-Caspase3) was upregulated, while anti-apoptotic factors, including Caspase3, Bcl2, and Bcl9, were downregulated (Figure 3E,F). From these results, it was indicated that the GdOFBAu potently inhibited tumor cell proliferation in vitro by suppressing the Wnt/beta-catenin pathway.

### 3.3. GdOFBAu Effectively Suppressed the Growth of Tumor In Vivo

**Figure 4:**
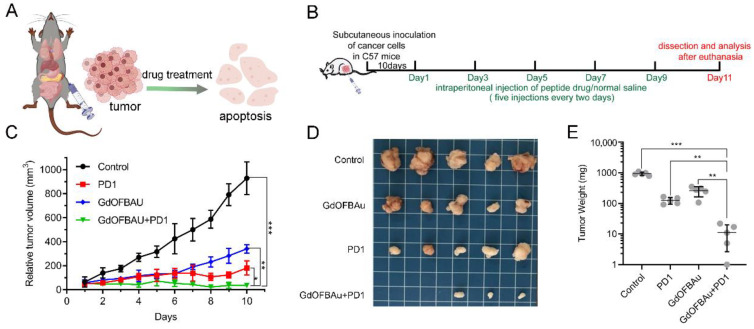
The suppressing effect of GdOFBAu combined with PD1 targeting for MC38 tumors in vivo. (**A**) Schematic diagram of intraperitoneal drug injection. (**B**) Schedule of the tumor vaccination and drug dosing. (**C**) Tumor growth curve of mice bearing MC38 treated with the indicated drug until the termination of the experiment. Photograph (**D**) and weight (**E**) of tumor mass stripped from euthanized mice bearing MC38. *p*-values were determined by student’s t-test: ***, *p* ≤ 0.05; **, *p* ≤ 0.1; *, *p* ≤ 0.5.

To examine the anticancer effect of GdOFBAu combined with anti-PD1 antibodies in vivo, MC38 cells with substantial β-catenin activation were subcutaneously inoculated into each female C57 mouse (n = 20). The dosing regimen is elaborated in Figure 4A,B. When the volume of the tumor reached approximately 50~100 mm^3^ (V = length × width^2^/2), four groups (n = 5) of MC38 tumor-bearing mice were allocated as: control (PBS), PD1, GdOFBAu and GdOFBAu + PD1 before treatment. All the mice received five doses of treatment, as indicated. As shown in Figure 4C, the growth of the tumor in the control group followed an upward trend and was almost uninhibited. Tumor growth was inhibited to a certain extent in both the PD1 and GdOFBAu treatment groups, while the combination of GdOFBAu and PD1 was the most effective, with a tumor suppression rate of over 90%. Moreover, the photograph (Figure 4D) and the average tumor weight (Figure 3E) stripped from the euthanized MC38 tumor-bearing mice were also consistent with the tumor volume reduction during dosing. Additionally, the result of H&E staining assays showed that a large number of dead cells were found in the GdOFBAu + PD1 group relative to other groups (Figure S2). Based on these results, it was concluded, therefore, that the combination therapy of the GdOFBAu and PD1 exerted better curative efficacy in comparison to drug monotherapy. 

### 3.4. GdOFBAu Enhanced the Tumor Response to Anti-PD1 Antibody Therapy In Vivo

**Figure 5:**
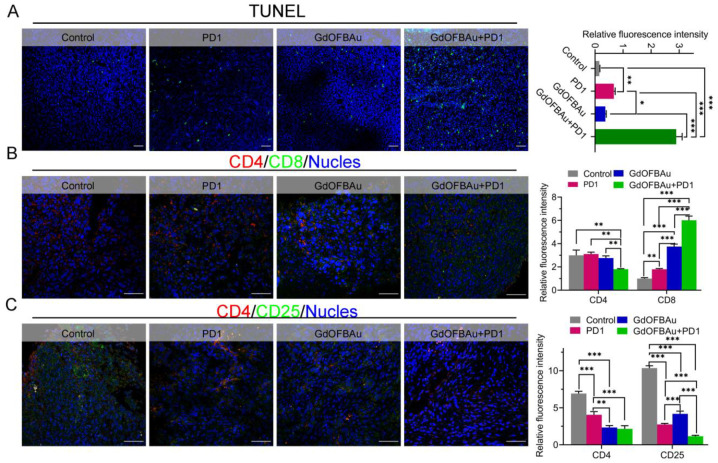
The synergy of GdOFBAu combined with PD1 in immunotherapy. (**A**) TUNEL staining of MC38 tumor mass after five drug administrations. Immunofluorescence of CD4^+^/CD8^+^ (**B**) and CD4^+^/CD25^+^ (**C**) in MC38 tumor after five drug administrations. Scale bar: 50 µm. *p*-values were determined by student’s *t*-test: ***, *p* ≤ 0.05; **, *p* ≤ 0.1; *, *p* ≤ 0.5.

It is well known that adequate infiltration of anti-tumor T cells in TME is essential for the success of cancer immunotherapy. It was reported that tumors infiltrated with high numbers of CD8^+^ T cells were more responsive to immunotherapy, and this was closely linked to cancer patient prognosis [50]. To further explore the underlying mechanism of enhanced immunotherapy efficacy provoked by GdOFBAu in combination with anti-PD1 antibodies, we detected the proportion of tumor-infiltrating T-cells (CD4^+^/CD8^+^, CD4^+^/CD25^+^) in tumors stripped from the euthanized mice after different treatments by immunofluorescence (IF) experiments. As expected, the counts of CD8^+^ showed a 1.5~3× increase in the drug combination group (GdOFBAu + PD1) compared with the monotherapy groups. Conversely, the drug combination group drastically reduced the CD25^+^ Treg cells, critical contributors to immunological escape (Figure 5A,B). These data suggest that GdOFBAu can sensitize tumors to immunotherapy by promoting Teff cell infiltration and activation while decreasing Treg cells. Moreover, the TUNEL staining results revealed that the highest rate of tumor apoptosis occurred in the combination group (Figure 5C). Taken together, when combined, the GdOFBAu synergized with anti-PD1 antibodies to enhance tumor immune response and prevent tumor progression.

### 3.5. GdOFBAu Showed Good Biosafety during Treatment

**Figure 6:**
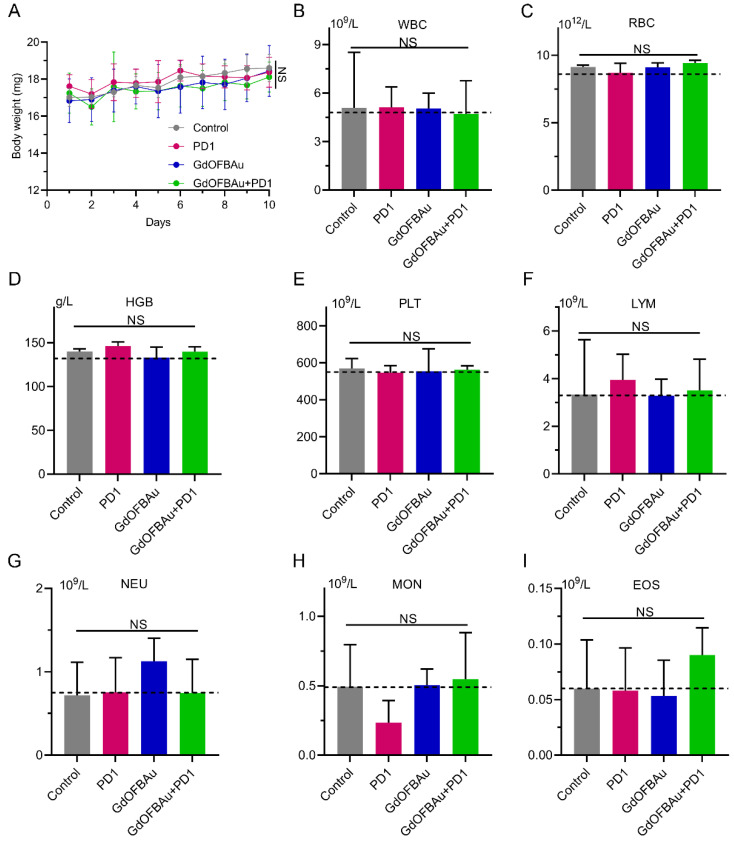
In vivo safety assessment of GdOFBAu. (**A**) Changes in the body weight in mice bearing MC38 during administration. (**B**–**I**) The indicators of blood routine cell test, including white blood cells (**B**), red blood cells (**C**), hemoglobin (**D**), platelets (**E**), lymphocytes (**F**), neutrophils (**G**), monocytes (**H**), and eosinophils (**I**).

**Figure 7:**
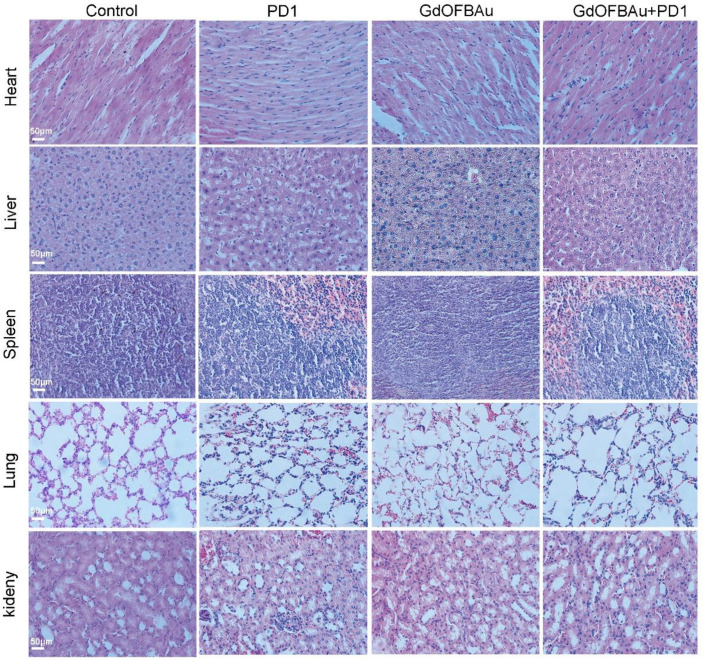
H&E images of organs (heart, liver, spleen, lung, kidney) stripped from euthanized mice bearing MC38 at the end of the experiment.

The toxic side effect of the anti-tumor drug in human internal organs and blood system is the main obstacle to druggability in the development of medicine. Therefore, biosafety is indispensable for the clinical translation of drug candidates. To evaluate the biosafety of the drug used in this study, we monitored the body weight change of mice-bearing MC38 during the 10 days of the dosing period. As shown in Figure 6A, compared to their initial weights, the weights of mice from each experiment group exhibited a slight fluctuating trend with no significant difference, indicating that the above-prepared drug delivery system had minimal systemic toxicity in vivo. The analysis of the hematologic index was further performed on mice blood plasma collected before euthanizing the mice (Figure 6B–I). The blood routine examination results showed no obvious changes among different treatment groups. In addition, there was no significant difference based on the results of the data. In addition, immunohistochemical analysis was applied to evaluate the toxicity of the injected drugs across major mice organs. No observably abnormal pathological alterations, such as inflammation or tissue necrosis, were observed in the hematoxylin and eosin (H&E)-stained and sliced mice organs after medical treatment (Figure 7), further demonstrating the excellent safety of GdOFBAu. Collectively, these above results reveal that GdOFBAu possesses favorable biosafety and biocompatibility in vivo application.

## 4. Discussion

Recently, there have been many reports about the importance of combination therapy to address the deficits of PD-1/PD-L1 blockade therapy, as only a minority of patients are responsive to this therapy. Since the abnormal activation of Wnt/β-catenin can promote immune escape, several targeting β-catenin/partners peptide inhibitors have been developed for boosting PD-1/PD-L1 blockade therapy. However, convenient, easily scalable, and drug-compatible drug delivery systems remain a challenge due to the drug barriers of peptides (poor cell membrane permeability and susceptibility to proteolytic degradation). In this study, a gadolinium–gold-based core–shell nanostructure Bcl9 peptide delivery system (GdOFBAu) was self-assembled with the mercapto group and metal–organic coordination and was able to inhibit tumor growth and improve the tumor’s susceptibility to PD-1 blockade therapy with high effectiveness and safety. This nanoparticle-based drug system may serve as a universal delivery tool for other drugs in combination with immunotherapy.

Nanoparticles of suitable size and shape are required for drug delivery. Our TEM image showed that GdOFBAu had a nano-star-like morphology with an average diameter of ~30 nm, which is a suitable size for tumor accumulation via the EPR effect. The irregular shape of GdOFBAu may be related to the ratio of reagents and reaction conditions; the Shen Mingwu group recently reported a Gd(OH)_3_@Au core/shell nano-star by varying the reaction time, solvent, and stabilizer [51]. In the future, we can try to change some conditions to develop new GdOFBAu with better properties. In vitro experiments showed that GdOFBAu inhibits MC38 proliferation through inhibition of the Wnt/beta-catenin pathway. After the GdOFBAu was used to treat the tumor and improve the anti-PD1 antibody immunotherapy, as expected, the GdOFBAu combined with the anti-PD1 antibodies showed great safety and efficacy. However, not all combination immunotherapy is safe. A PpNF (Dox) nanocage developed by the SoyounKim group decreased the weight of tumor-bearing mice in combination immunotherapy [52], which might be attributed to the off-target toxicity of Dox as it was effectively delivered to mice bodies by the nanocage. In view of this issue, we used a Bcl9 peptide in co-immunotherapy with intrinsic properties as we described. In terms of the mechanism, some groups reported that inhibition of Bcl9 can overcome resistance to immune checkpoint blockades by decreasing Treg cells and increasing the CD8^+^ T cells in CRC [53,54]. Inspired by it, we detected the counts of CD4^+^/CD8^+^ and CD4^+^/CD25^+^ in the tumors and found that GdOFBAu indeed promoted the tumor infiltration of Teff cells (CD8^+^) and decreased the Treg cells (CD25+), further confirming that using GdOFBAu to target Bcl9/β-catenin might be a potentially efficient strategy for combination immunotherapy. Although some success was achieved in peptide delivery, there is still a long way to go before clinical translation can be achieved in the future.

## 5. Conclusions

In summary, to overcome the pharmaceutical obstacle of peptides in sensitizing cancer immunotherapy, a gadolinium–gold-based core–shell nanostructure drug delivery system was established. In our design, the Bcl9 peptide was incorporated into the gadolinium–gold core–shell nanostructure to form GdOFBAu via self-assembly triggered by mercapto group coordination and metal–organic coordination. As expected, the synergistic effect of GdOFBAu combined with PD1 had a synergistic effect in inhibiting the tumor growth and enhancing the response to immune therapy, which not only induced the apoptosis of cancer cells, but also promoted the tumor infiltration of Teff cells (CD8^+^) and decreased the Treg cells (CD25^+^). More importantly, the GdOFBAu exhibited favorable biosafety and biocompatibility in vivo. Collectively, this system seems to provide a promising platform for delivering anticancer peptides to sensitize cancer immunotherapy with high tumor specificity and safety.

## Data Availability

Not applicable.

## Supplementary Materials

The following supporting information can be downloaded at: https://www.mdpi.com/article/10.3390/pharmaceutics14061306/s1, Figure S1: FESEM picture of GdOFBAu nanoparticle, Figure S2: He staining of tumor stripped from euthanized mice bearing MC38 at the end of experiment.
