# Supplementary material for: Turning a Targeting β-Catenin/Bcl9 Peptide Inhibitor into a GdOF@Au Core/Shell Nanoflower for Enhancing Immune Response to Cancer Therapy in Combination with Immune Checkpoint Inhibitors
Source: Pharmaceutics. 2022 Jun 20;14(6):1306. doi: 10.3390/pharmaceutics14061306 (PMC9228893; doi:10.3390/pharmaceutics14061306)
Supplement: Supplementary file 1 [file pharmaceutics-14-01306-s001.zip › pharmaceutics-1688704-supplementary.pdf]

Supporting Information

Turning a targeting β-catenin/Bcl9 peptide inhibitors into a GdOF@Au core/shell nanoflower for enhancing immune response to cancer therapy in combination with immune checkpoint inhibitors

Weiming You[1,2], Fang Ma[1,2], Zhang Zhang [3,*], Jin Yan [1,2,*]

1. National & Local Joint Engineering Research Center of Biodiagnosis and Biotherapy, The Second Affiliated Hospital of Xi'an Jiaotong University, Xi'an 710004, China.

2. Department of Tumor and Immunology in precision medical institute, Western China Science and Technology Innovation Port, Xi'an 710004, China

3. General Surgery Department, Tang Du Hospital, Fourth Military Medical University, 710032 Xi'an, Shaanxi, China.

[*] Corresponding authors:

Email: yanjin19920602@xjtu.edu.cn (J. Yan)

Email: zhangz0613@163.com (Z. Zhang)

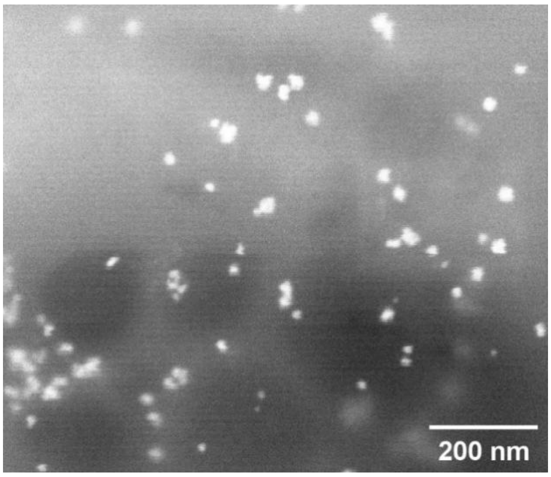

Figure S1. FESEM picture of GdOFBAu nanoparticle.

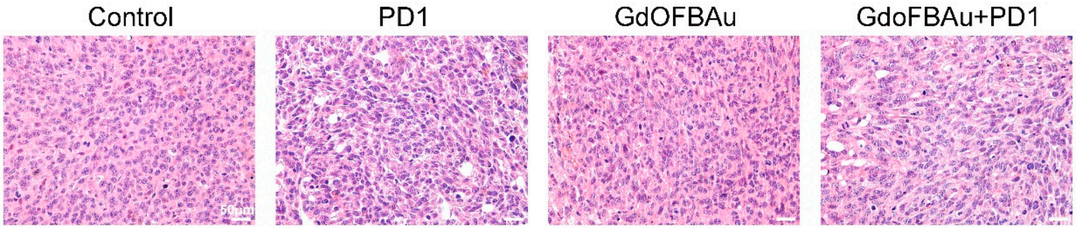

Figure S2. He staining of tumor stripped from euthanized mice bearing MC38 at the end of experiment.
